# Bioinformatics and experimental studies of anti-leukemic activity from 6-gingerol demonstrate its role in p53 mediated apoptosis pathway

**DOI:** 10.17179/excli2019-2008

**Published:** 2020-05-06

**Authors:** Chawalit Chatupheeraphat, Chanin Nantasenamat, Kamolchanok Deesrisak, Sittiruk Roytrakul, Usanarat Anurathapan, Dalina Tanyong

**Affiliations:** 1 Department of Clinical Microscopy, Faculty of Medical Technology, Mahidol University, Nakhon Pathom 73170, Thailand; 2 Center of Data Mining and Biomedical Informatics, Faculty of Medical Technology, Mahidol University, Bangkok 10700, Thailand; 3 Proteomics Research Laboratory, National Center for Genetic Engineering and Biotechnology, 113 Thailand Science Park, Klongluang, Pathumthani 12120, Thailand; 4 Department of Pediatrics, Faculty of Medicine, Ramathibodi Hospital, Mahidol University, Bangkok 10400, Thailand

**Keywords:** 6-gingerol, leukemia, apoptosis, network pharmacology, p53, bioinformatics

## Abstract

6-gingerol is a traditional medicine that possesses anti-cancer activity against several types of cancer. However, the mechanism of action still remains unclear. Therefore, this study explored the effects of 6-gingerol on anti-leukemic mechanisms in NB4, MOLT4, and Raji leukemic cell. Results indicated that 6-gingerol inhibited cell proliferation and induced cell apoptosis in these 3 cell lines. Moreover, 6-gingerol was shown to increase the mRNA expression of the caspase family thereby suggesting that 6-gingerol induced apoptosis through the caspase-dependent pathway. To explore the signaling pathway regulating 6-gingerol induced apoptosis, we utilized and integrated the network pharmacology approach together with experimental investigations. Targets of 6-gingerol were identified from ChEMBL and STITCH databases, which were used for constructing the protein-protein interaction (PPI) network. Results from the PPI network indicated that p53 was a key regulator. Moreover, it was found that 6-gingerol could increase the levels of p53 mRNA in all leukemic cell lines. Thus, 6-gingerol has shown to have anti-cancer activity. In addition, p53, BAX and BCL2 could be involved in the apoptosis pathway of these leukemic cells. This study is anticipated to be useful for the development of 6-gingerol as an anti-leukemic drug in the future.

## Introduction

Acute leukemia is characterized by the uncontrolled proliferation and impaired differentiation of hematopoietic stem cells during hematopoiesis (Rose-Inman and Kuehl, 2014[27]). Treatment strategies relying on chemotherapy in both myeloid and lymphoid leukemia have shown unsatisfactory outcome along with disease complications (Liesveld and Lichtman, 2016[20]; Niederhuber et al., 2013[22]; Salzer et al., 2010[28]). Moreover, conventional chemotherapy is associated with various side effects (i.e., ranging from mild to severe) encompassing nausea, cardiac and renal toxicity and myelosuppression (Eiring et al., 2011[11]; Kell, 2004[15]). Therefore, there is an urgent need to seek novel therapeutic agents with less side effects. Recently, many research works have focused on natural products which have shown potential effects in regards to prevention and treatment (Aggarwal and Shishodia, 2006[2]; Burton, 2003[5]; Kizaki, 2006[16]; Weng and Yen, 2012[31]). Furthermore, natural products have exhibited their actions against multiple targets via various pathways which are able to achieve the desired effects for disease treatment (Wu et al., 2013[32]).

Ginger (*Zingiber officinale*) has been used in both culinary and traditional medicine for relieving many symptoms consisting of inflammation, asthma, stroke, rheumatic disorders and gastrointestinal discomforts (Afzal et al., 2001[1]). The rhizome contains 6-gingerol, which is the most abundant phenolic compound that exhibits anti-cancer activities against several cancers such as colon cancer, breast cancer, skin cancer, and myeloid leukemia. Several studies suggest that 6-gingerol modulates apoptosis, cell cycle regulation, oxidative stress and metastasis inhibition by means of various signaling pathways including miRNA27b, MAPK/AP-1, MMP9 and Cyclins (Lee et al., 2008[17]; Nigam et al., 2009[23]; Radhakrishnan et al., 2014[25]; Rastogi et al., 2014[26]). However, only few studies have elucidated the mechanism of 6-gingerol in inducing cell death in leukemia (Rastogi et al., 2014[26]; Wang et al., 2003[30]).

Network pharmacology is a promising bioinformatic approach for gaining systemic insights into the molecular mechanism on the interactions between drugs and their targets as well as their implications in diseases at the holistic level (Hopkins, 2008[13]; Zeng and Yang, 2017[35]). On the basis of this context, network pharmacology can be used to explore the underlying mechanism of diseases and for studying the effect of drugs in the signaling pathways. Chen et al. utilized network pharmacology for revealing that JUN plays an important role in the mechanism of Yinchensini decoction (i.e., a classical traditional Chinese medicine) (Chen et al., 2018[7]). Moreover, the network pharmacology approach was also used for studying the mechanism of action of Temozolomide (TMZ), a second-generation imidazotetrazine lipophilic prodrug, in glioblastoma (Xu et al., 2018[33]). Furthermore, network pharmacology had also been used to study the mechanism of action of drugs in leukemia. The MAPK pathway was identified as an important pathway in the molecular mechanism of action of Qingdai on CML (Li et al., 2018[18]). However, the mechanism of 6-gingerol remains unclear and has not yet been elucidated by network pharmacology.

In this article, we studied the effect of 6-gingerol on leukemic cell lines as compared to PBMC (i.e. normal white blood cells) for examining the cell death mechanism. Through the use of network pharmacology, we have revealed key proteins involved in 6-gingerol induced cell death. This research represents the first study to investigate the mechanism of 6-gingerol on leukemic cell lines through the integrative use of network pharmacology along with experimental validations.

## Materials and Methods

### Chemicals and reagents

6-gingerol and propidium iodide (PI) were purchased from Sigma Aldrich (St. Louis, CA, USA). Briefly, 6-gingerol was dissolved in DMSO as a 34 mM stock solution and stored at -20 °C. 3-(4-dimethylthiazol-2-yl)-2-5-diphenyl tetrazolium bromide (MTT) was purchased from Invitrogen (Walthem, MA, USA). Annexin V assay kit was purchased from BD Biosciences (Palo Alto, CA, USA). Ultra-pure water was used for the preparation of reagents as well as in all experiments. 

### PBMC collection and preparation

Sample collection in this study was conducted according to “The Code of Ethics of the World Medical Association (Declaration of Helsinki)” for experiments involving humans. Specimen collection and experimental protocols for handling clinical samples were approved by the Committee on Human Rights Related to Research Involving Human Subjects, Faculty of Medicine, Ramathibodi Hospital, Mahidol University (MURA2019/778). Written informed consents were obtained from participants prior to blood collection. 10 ml of blood was collected from each healthy volunteer via venous phlebotomy. PBMC was separated by Lymphoprep (Alere Technologies AS, Oslo, Norway) following manufacturer’s protocol. Briefly, the blood was diluted with phosphate buffer saline (PBS) in a 1:1 ratio. Lymphoprep was added into 50 ml conical tubes, and the diluted blood was gently layered on top of the Lymphoprep. The sample tubes were then centrifuged at 800 g for 20 min without declined acceleration. Lastly, the PBMC layer was collected and washed twice with medium before being used for further experiments.

### Leukemic cell culture 

NB4 (acute promyelocytic leukemia cell line) and MOLT4 (acute T-lymphocytic leukemia cell line) were obtained from Cell line services (Eppelheim, Germany). Raji (B-lymphoma cell line) was kindly gifted from Asst. Prof. Dr. Usanarat Anurathapan (Faculty of Medicine, Ramathibodi Hospital, Mahidol University). Leukemic cells were cultured in RPMI1640 medium supplemented with 10 % (v/v) fetal bovine serum (FBS) and 100 U/ml penicillin and 100 μg/ml streptomycin (Gibco Life Technologies, Walthem, MA, USA). The cells were maintained in an incubator containing humidified atmosphere of 5 % CO_2_ and the media was changed twice a week.

### Cell viability by MTT assay

Briefly, 15,000 cells were seeded in a 96-well plate and treated with 100, 200, and 300 µM of 6-gingerol for 24 and 48 hours. After which, 5 mg/ml of MTT was added to each well and incubated for 4 hours. Formazan crystals were solubilized by adding 10 % sodium dodecyl sulfate (SDS) in 0.01 M HCl and incubated overnight. The developed color was measured as optical density (OD) using a microplate reader at 570 nm with GEN5^TM^ analysis software (BioTek Instruments, Inc., Winooski, VT, USA). All experiments were done in triplicate. The results were expressed as a percentage of cell viability as compared with the control, and the IC_50_ was calculated.

### Apoptosis assay by Annexin V/PI staining 

Apoptosis was determined by using the Annexin V assay kit (BD Biosciences, Palo Alto, CA, USA) following the manufacturer’s protocol. Briefly, leukemic cells (2x10^5^ cells) were treated with 200 µM of 6-gingerol for 48 hours. After the incubation time, cells were harvested and washed twice with phosphate buffer saline (PBS). Then, cells were stained with 5 µl of Annexin V-FITC and 5 µl of 50 µg/ml propidium iodide (PI). The apoptotic cells were determined by using FACSCantoII flow cytometer and analyzed via FACSDiva software (BD Biosciences, Palo Alto, CA, USA). All experiments were conducted in triplicate.

### Cell cycle assay by flow cytometry

Leukemic cells (2x10^5^ cells) were treated with 200 µM of 6-gingerol for 48 hours. Cells were harvested and washed twice with PBS. Then, the cells were fixed dropwise with 70 % ethanol and incubated overnight at -20 °C. The fixed cells were washed with PBS and incubated with 100 µg/ml RNase A at 37 ^o^C for 30 min. 50 µg/ml of propidium iodide (PI) was then added and incubated for an additional 30 min in the dark, at room temperature. The cells were examined by FACSCantoII flow cytometer and analyzed using the FACSDiva software (BD Biosciences, Palo Alto, CA, USA). All experiments were conducted in triplicate.

### Quantitative Real-Time Polymerase Chain Reaction (qRT-PCR) to determine the level of gene expression

Leukemic cells (2x10^5^ cells) were treated with 200 µM of 6-gingerol for 48 hours. Total RNA was extracted by Genezol^®^ (New England Biolab, Inc., Ipswich, MA, USA) following manufacturer’s procedure. The RNA concentration was determined by nanodrop2000 (Thermo Scientific, Waltham, MA, USA). After which, 2 µg of RNA was reverse-transcribed using the RevertAid first strand cDNA synthesis kit (Thermo Scientific, Waltham, MA, USA). Next, real time PCR was performed using the designed primers. The sequences of primers used in this study are shown in Table 1(Tab. 1). The reactions were carried out with Luna^®^ real time PCR master mix (New England Biolab, Inc., Ipswich, MA, USA) in the bio-rad thermal cycle CX1000 (Bio-Rad, Inc., Hercules, CA, USA). Gene expression levels were determined with the 2^-∆∆ct^ technique, and levels were normalized using GAPDH as the house keeping gene. 

### Construction of target database of 6-gingerol

The targets of 6-gingerol were obtained from two sources. The first source was the STITCH database (http://stitch.embl.de/), which provided the interaction between molecule and its molecular partner. The keyword “6-gingerol” was used as a search parameter with a confidence score of >0.4. This produced a list of 9 target proteins as shown in Supplement S1 (*List of target proteins from STITCH*). The second source was composed of using the molecular similarity approach since similar chemical structures of compounds may share common target proteins. These similar compounds were identified from the ChEMBL database (https://www.ebi.ac.uk/chembl/) by using a similarity threshold of >70 % for obtaining compounds with a similar chemical structure to that of 6-gingerol. The bioactivity of these compounds was then obtained. Target proteins of identified compounds affording bioactivity potency with a concentration lesser than 100 µM were used as a threshold for filtering potent compounds as shown in Supplement S2 (*List of target proteins from ChEMB*L).

### 6-gingerol related protein-protein interaction network construction

The compiled list of target proteins against 6-gingerol obtained from ChEMBL and STITCH databases and the caspase family proteins, resulted in a total of 24 proteins as listed in Table 2(Tab. 2).

The protein-protein interaction (PPI) network was constructed by importing all potential target proteins to the STRING database (http://string-db.org), which is a protein-protein interaction database. In order to obtain an optimal network configuration, an intermediate confidence score of >0.4 was used. Next, the first and second spheres of interaction were varied in the range of 10-100 nodes. All resulting data from STRING were imported to and visualized in Cytoscape, version 3.7.1. The biological network is a scale free network which follows the power law of P(k)~k-^γ^ with a distribution degree (Broido and Clauset; 2019[4]). All networks that corresponded with γ and R-squared in correlation with the power law, were built and computed in Cytoscape. Furthermore, the γ, R-square and correlation coefficient of all networks were listed as Supplement S3 (*Data of network topolog*y). In addition, the network with 30 first interaction spheres and 40 second interaction spheres having a lower value of γ and a higher value of R-square, cluster coefficient and correlation coefficient for both indegree and outdegree was selected for further experiments.

### Gene ontology (GO) and KEGG pathway enrichment analysis of PPI

DAVID was used to identify significant groups of genes and pathways that are related to the data set used in this study. All 92 proteins in the network were imported to the DAVID Bioinformatics resource 6.8. (https://david.ncifcrf.gov/) for pathway annotation and gene ontology analysis (Huang et al., 2009[14]). Generated files of GO biological processes, GO molecular function, GO cellular component and KEGG pathway analysis were obtained. The pathway or gene ontology and KEGG pathways which have <0.01 Bonferroni p-value were selected.

### Statistical analysis

Results were expressed as mean ± SEM. All statistical analyses were performed using GraphPad Prism 6 (GraphPad Inc., San Diego, CA, USA). Experiments containing only two groups were analyzed using the student’s t-test whereas experiments containing more than two groups were analyzed using one-way ANOVA with p-value <0.05 being considered as statistically significant.

## Results

**Figure 1:**
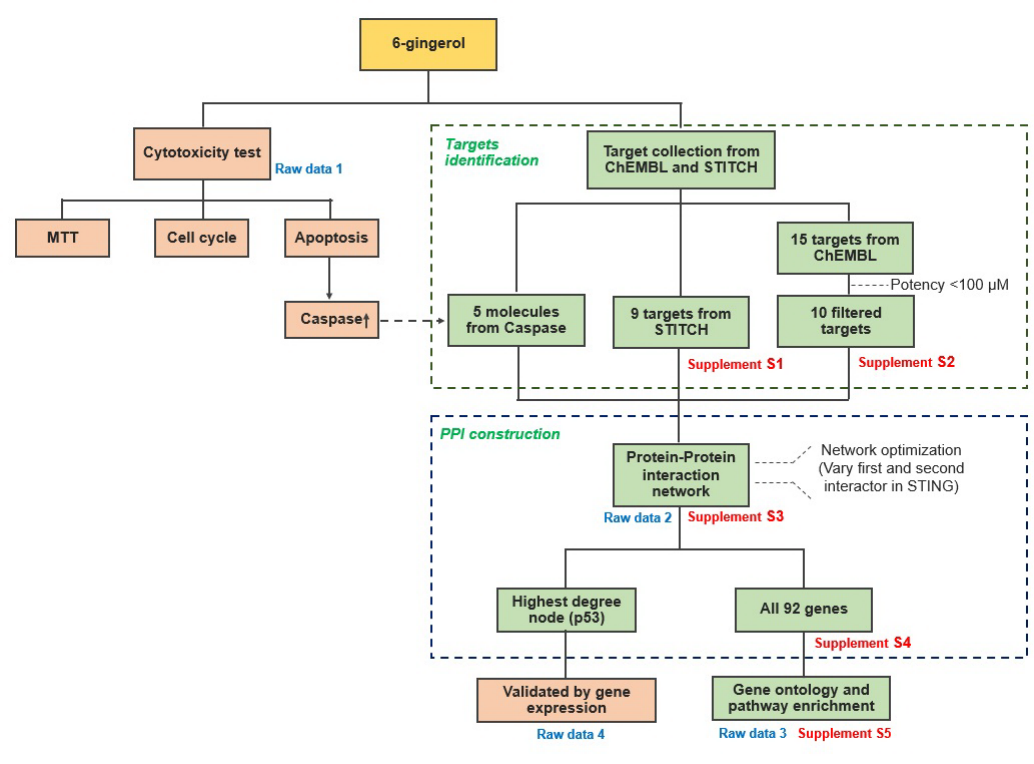
Schematic overview of experimental and computational methodologies employed in this study

The conceptual framework of this study is summarized in Figure 1(Fig. 1). Briefly, we are investigating the anti-leukemic activity of 6-gingerol by means of computational and experimental approach and this encompasses the use of several bioinformatics databases and tools for shedding light on putative target proteins that interact with 6-gingerol to give rise to anti-leukemic activity via the induction of the apoptosis pathway.

### Cytotoxic effect of 6-gingerol on acute leukemic cell lines

**Figure 2:**
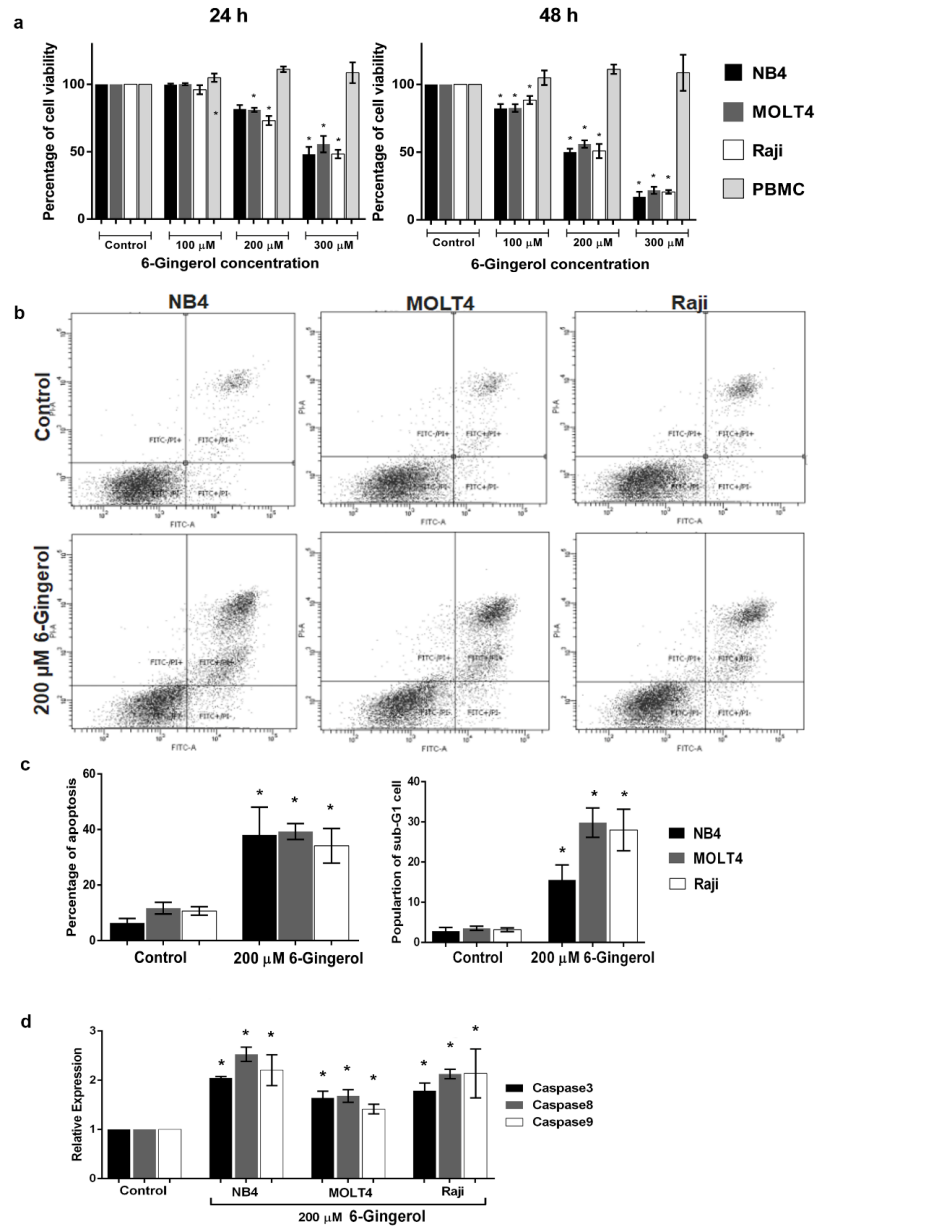
Effect of 6-gingerol on cell viability and apoptosis in leukemic cell lines (i.e., NB4, MOLT4 and Raji) and PBMC. (a) Cell viability of leukemic cell lines and PBMC treated with 6-gingerol at various concentrations for 24 h (left panel) and 48 h (right panel). Leukemic cell lines treated with 200 µM of 6-gingerol were subjected to (b) cytometric analysis of apoptosis, (c) summary of the percentages of apoptosis (left panel) and sub-G1 cell cycle assay (right panel) and (d) mRNA expression level of caspase family genes (i.e., caspase 3, 8 and 9). All data are shown as mean±SEM, n=3 and *p<0.05 were considered to be statistically significant from the control.

To determine the effect of 6-gingerol induced leukemic cell death, we observed the cell viability of leukemic cell lines under 6-gingerol treatment. As presented in Figure 2a(Fig. 2), 6-gingerol was found to significantly decrease the cell viability in a dose- and time-dependent manner. The IC_50_ values of 6-gingerol against NB4, MOLT4 and Raji leukemic cell lines were 313±32, 338±4 and 297±18 µM, respectively, at 24 hours while the IC_50_ values were 194±12, 208±6 and 204±8 µM, respectively, at 48 hours. For the non-cancerous cells (i.e., PBMC), no effect on cell viability was observed. The results suggest that 6-gingerol showed toxicity in leukemic cells without toxicity to PBMC.

### Apoptosis induction of 6-gingerol on acute leukemic cell lines

To evaluate the role of 6-gingerol in apoptosis, we measured the apoptotic cells at a 6-gingerol concentration of 200 µM. After 48 hours of incubation, 6-gingerol increased the level of apoptosis in NB4, MOLT4 and Raji cell lines, with values of 38.07±10.02 %, 39.27±2.84 % and 34.15±6.22 %, respectively (p<0.05, Figure 2b(Fig. 2)). Furthermore, the results of apoptosis assay were also seen to correlate with that of the cell cycle assay. Particularly, 6-gingerol increased the proportion of sub-G1 phase by 15.6 %, 29.8 % and 28.0 % in NB4, MOLT4 and Raji cell lines, respectively, as shown in Figure 2c(Fig. 2) (p<0.05). These results infer that 6-gingerol could induce cell apoptosis in acute leukemic cell lines.

### Effect of 6-gingerol on expression levels of caspase gene family

We further investigated the molecular mechanism of apoptosis as induced by 6-gingerol. After 48 hours of treatment with 6-gingerol at the IC_50_ concentration, results showed that 6-gingerol upregulated caspase 3, caspase 8 and caspase 9 mRNA expression levels in these cell lines (p<0.05, Figure 2d(Fig. 2)). This indicates that 6-gingerol induced leukemic cell apoptosis through a caspase-dependent apoptosis pathway.

### Construction of 6-gingerol related protein-protein interaction network

**Figure 3:**
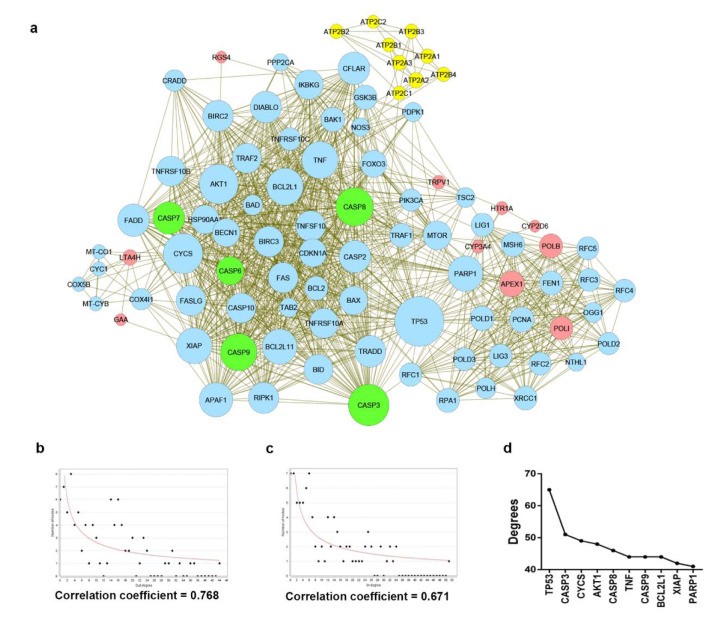
The protein-protein interaction network modulated by 6-gingerol. (a) The interaction network imported from Cytoscape, where nodes represent the protein (i.e. yellow, pink, and green nodes represent the searched proteins from STITCH, ChEMBL, and Caspase family respectively, blue nodes represent the other proteins that participated in the network) and edges represent the interaction. The size of nodes varies upon the degree of the node. (b) and (c) The network topology depicted with correlation coefficient, (b) the in-degree distribution and (c) out-degree distribution. (d) Number of degrees calculated based on the interaction network. The top 10 highest degree nodes were shown.

Next, we searched for possible targets of 6-gingerol using the ChEMBL and STITCH databases. 10 targets were obtained from ChEMBL and 9 targets were obtained from STITCH as shown in Table 2(Tab. 2). The obtained target proteins together with the caspase family proteins were used as the key in the STING database for obtaining relevant information of protein interactions. The interacting data were introduced into Cytoscape to visualize protein–protein interaction networks of 6-gingerol. The PPI network contained a total of 1072 interactions (i.e., the edges connecting the nodes) involving 92 proteins (i.e., the nodes). All 92 proteins in the network and their topology parameters are shown in Supplement S4 (*All molecules of network*). It should be noted that yellow, pink and green nodes represent proteins derived from the STITCH, ChEMBL and Caspase searched proteins, respectively (Figure 3a(Fig. 3)). Moreover, it should be noted that the size of the node indicates the number of edges interacting with that node (i.e., higher number of interactions presented to a node result in a larger sized node). Topology parameters of the network were calculated using the Network Analyzer as implemented in Cytoscape. The probability of out- and in-degree distribution P(k) followed the power law scaling behavior, which has a degree exponent of 0.512 and 0.526 with corresponding correlation coefficient values of 0.768 and 0.671, respectively and R-square values of 0.433 and 0.512, respectively (Figure 3b and 3c(Fig. 3)). The small value of degree exponent (<2) indicates that the network is a scale-free network, which is a characteristic signature of biological networks (Broido and Clauset, 2019[4]). It can be observed from the results that p53 was the highest degree containing node (Figure 3d(Fig. 3)). Previous reports suggest that a high degree node (hub) exerts a strong influence in controlling the topology of the network (Babu et al., 2004[3]; Döhner et al., 2010[10]). Therefore, it can be hypothesized that p53 plays an important role in the induction of apoptosis in leukemic cells with the use of 6-gingerol. 

### Gene ontology (GO) and KEGG pathway enrichment analysis of 6-gingerol related PPI

**Figure 4:**
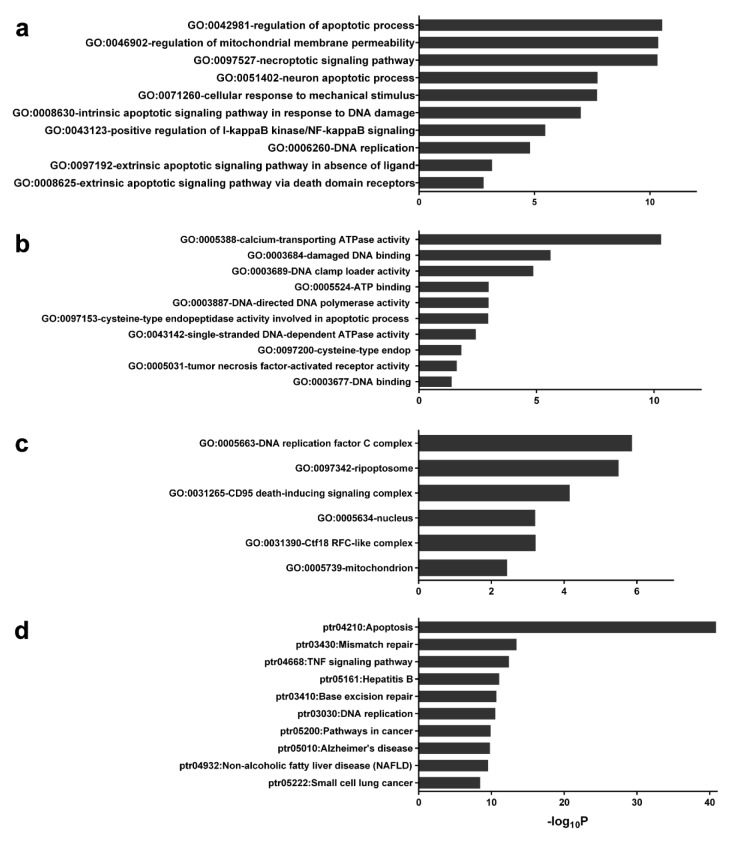
Gene ontology annotation and KEGG related pathway of protein in the network. The gene ontology and KEGG pathways that had Bonferrini p-value lower than 0.01 were obtained from DAVID. a: Top 10 GO Biological property, b: Top 10 GO Molecular functions, c: Top 6 GO Cellular property and d: Top 10 KEGG related pathway.

GO annotation and enrichment of network protein in three aspects including, biological process (BP), molecular function (MF) and cellular component (CC) was analyzed in DAVID. The generated files are shown in Supplement S5 (*GO and KEGG data from DAVID*). A total of 102 GO_BP pathways were obtained which consisted of 20 significant pathways. The top 10 significantly enriched biological processes comprised extrinsic apoptotic signaling pathway, DNA replication, positive regulation of I-kappaB kinase/NF-kappaB signaling, intrinsic apoptotic signaling pathway in response to DNA damage and others as shown in Figure 4a(Fig. 4). Furthermore, a total of 27 GO_MF were obtained which consists of 12 pathways of significance. The top 10 significantly enriched molecular functions were calcium-transporting ATPase activity, damaged DNA binding, DNA clamp loader activity, ATP binding and others as shown in Figure 4b(Fig. 4). In addition, a total of 22 GO_CC were obtained which had only 6 components with significance. All significant cellular components were DNA replication factor C complex, ripoptosome, death-inducing signaling complex, nucleus, Ctf18 RFC-like complex and mitochondrion as shown in Figure 4c(Fig. 4). Similarly, from a total of 77 KEGG pathways that had been obtained, 12 of these pathways were found to be significant. 

The top 10 significant pathways were composed of Apoptosis, Mismatch repair, TNF signaling pathway, Hepatitis B, Base excision repair and others as shown in Figure 4d(Fig. 4).

### Effect of 6-gingerol on mRNA expression of p53 pathway

**Figure 5:**
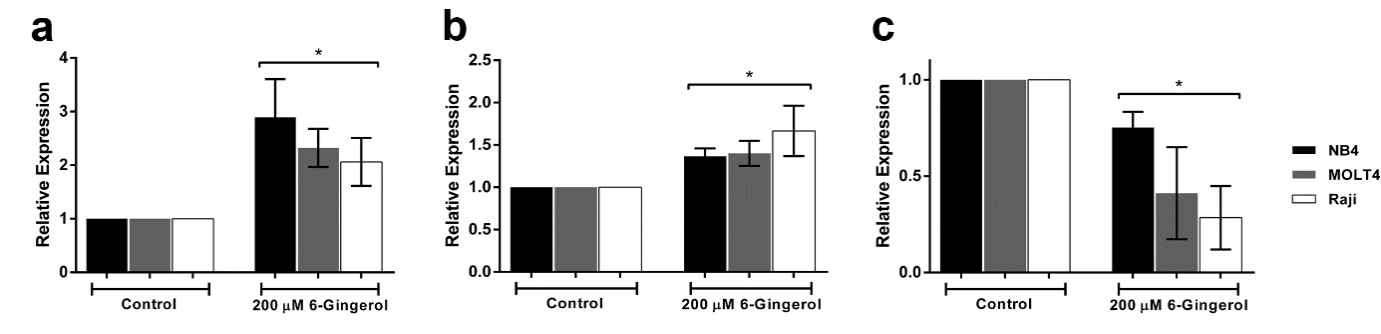
Effect of 6-gingerol on mRNA expression levels of (a) p53, (b) BAX and (c) BCL2 in leukemic cell lines treated at IC_50_ dose of 200 µM. All data are shown as mean ± SEM, n=3 and *p<0.05 were considered to be statistically significant from the control.

To validate results from the network analysis, we examined the mRNA expression levels of p53 and related genes by quantitative real-time PCR. The mRNA expression levels of p53 and BAX were shown to increase in the 6-gingerol treated group as compared with the control group. While, BCL-2 was shown to decrease in the 6-gingerol treated group when compared with the control group (p<0.05, Figure 5(Fig. 5)). Therefore, it can be inferred that 6-gingerol induces leukemic cell apoptosis via the p53 pathway.

For more information see Supplementary data 1 for Figure 2, Supplementary data 2 for Figure 3, Supplementary data 3 for Figure 4, Supplementary data 4 for Figure 5.

## Discussion

Acute leukemia is a cancer involved in hematopoiesis. Over the last two decades, the treatment and prognosis of acute leukemia in patients have significantly improved. However, the risk of treatment-related mortality associated with chemotherapy and other side effects are a great cause of concern. In acute myelogenous leukemia, the mortality associated with chemotherapy is dependent on the age as well as underlying diseases which limit tolerance against the side effects of chemotherapy (Döhner et al., 2010[10]). The mortality rate was seen to increase to 60 % in patients older than 70 years (Parikh et al., 2016[24]). On the contrary, in patients with acute lymphoid leukemia, conventional treatments in children show an overall survival rate of around 80 %. However, the story is much different for adults whereby the chance of reaching complete remission is only approximately 30 to 40 % (Niederhuber et al., 2013[22]). Nowadays, alternative treatments using natural compounds or herbs have also been studied (Demain and Vaishnav, 2011[9]). 

Ginger (*Zingiber officinale*), a spice that has been used extensively in culinary and recognized to afford several bioactivities, exhibits anti-cancer properties against breast, skin and colon cancers (de Lima et al., 2018[8]). The aqueous extract from ginger showed anti-oxidant and anti-cancer activity against human breast cancer cell lines via p53 and caspase 9 induction. Moreover, the extract showed a synergetic effect when administrated in combination with tamoxifen (Vemuri et al., 2017[29]). In the ginger constituents, 6-gingerol was identified as a major pungent component in fresh ginger rhizome where it exhibited a wide range of bioactivities such as anti-inflammatory, analgesic and cardiotonic effects (Chang and Kuo, 2015[6]; Young et al., 2002[34]). Several studies have confirmed the anti-cancer activity of 6-gingerol. Particularly, Nigam et al. (2009[23]) showed that 6-gingerol increased the reactive oxygen species thereby inducing the mitochondrial cell death pathway in the A431 skin cancer cell line (Nigam et al., 2009[23]). Furthermore, 6-gingerol was found to inhibit colon cancer cell lines via apoptosis induction by MAPK/AP-1 inhibition and G2/M cell cycle arrest mechanism (Lin et al., 2012[21]; Radhakrishnan et al., 2014[25]). Moreover, 6-gingerol had also shown anti-metastasis activity in MDA-MB-231 breast cancer cell line through MMP2 and MMP9 inhibition (Lee et al., 2008[17]).

In this study, we used an integrated network pharmacology and experimental approach to reveal the possible mechanism of 6-gingerol and its targets in leukemic cell lines. We identified the most probable genes that participate in the 6-gingerol induced leukemia apoptosis network to be the p53 signaling pathway. In addition, results from gene ontology and KEGG pathways showed that apoptosis was the key mechanism in the 6-gingerol network. GO:0008625 (i.e., the extrinsic apoptotic signaling pathway via death domain receptors) and GO:0097192 (i.e., the extrinsic apoptotic signaling pathway in absence of ligand) were the top 2 results for molecular property gene ontology that correlated with the significant result in gene ontology molecular function. Similarly, GO:0003684 (i.e., damaged DNA binding), GO:0097153 (i.e., cysteine-type endopeptidase activity involved in apoptotic process) and GO:0097200 (i.e., cysteine-type endopeptidase activity involved in the execution phase of apoptosis) were ranked 2, 6 and 8 in the gene ontology molecular function, respectively. Furthermore, results from the KEGG pathway analysis showed that the apoptosis pathway was the most highly connected and significant pathway in the network. These results suggested that the mechanism of action of 6-gingerol was mediated by apoptosis as induced by the p53 pathway. Moreover, the experimental results confirmed our bioinformatic investigation in which the MTT assay proved that 6-gingerol could inhibit growth and proliferation of leukemic cells. Finally, quantitative real-time PCR also validated the bioinformatic result where 6-gingerol was able to increase the levels of p53 and BAX gene expression whereas, the level of BCL2 was reduced. 

Several studies have reported that the p53 signaling pathway involved in apoptosis can be induced by natural compounds. Erianin, a compound from *Dendrobium chrysotoxum Lindl*, showed anti-cancer activity against human cervical cancer cell lines via p53 regulation (Li et al., 2018[19]). In addition, resveratrol increased the phosphorylated p53 at serine 20 to induce apoptosis in MCF-7 cell line (Hernandez-Valencia et al., 2018[12]). Moreover, Lin et al. (2012[21]) also reported that 6-gingerol could induce cell cycle arrest in colon cancer cell line via the p53 pathway.

## Conclusions

The present study revealed that 6-gingerol exhibits anti-leukemic activity in leukemic cell lines by reducing cell viability, activating apoptosis and increasing caspase 3 expression. Furthermore, the results from our bioinformatic approach provided a possible mechanism of action induced by 6-gingerol as the p53-dependent pathway. Moreover, the experimental validation of the bioinformatic results also highlighted the upregulation of p53, BAX and downregulation of BCL2. These results indicate that 6-gingerol induces apoptosis in leukemic cell lines via the p53-dependent pathway. Thus, this study reveals the significance of 6-gingerol which could be developed as a candidate drug for the treatment of leukemia in the future.

## Conflict of interest

The authors declare no conflict of interest.

## Acknowledgements

This project was supported by the Royal Golden Jubilee Ph.D. Scholarship (No. PHD00162560) under Thailand Research Fund (TRF) and the TRF Research Career Development Grant (No. RSA6280075) from the Thailand Research Fund, the Office of Higher Education Commission and Mahidol University.

## Supplementary Material

Supplement S1

Supplement S2

Supplement S3

Supplement S4

Supplement S5

Supplementary data 1 for Figure 2

Supplementary data 2 for Figure 3

Supplementary data 3 for Figure 4

Supplementary data 4 for Figure 5

## Figures and Tables

**Table 1:**
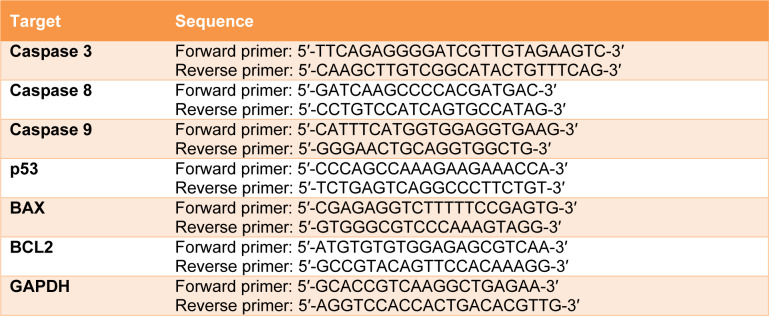
Primers used in this study

**Table 2:**
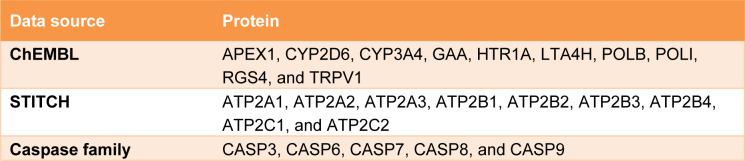
Target proteins used in the construction of the protein-protein interaction network
